# Preparation and Characterization of Gradient Compressed Porous Metal for High-Efficiency and Thin-Thickness Acoustic Absorber

**DOI:** 10.3390/ma12091413

**Published:** 2019-04-30

**Authors:** Xiaocui Yang, Xinmin Shen, Panfeng Bai, Xiaohui He, Xiaonan Zhang, Zhizhong Li, Liang Chen, Qin Yin

**Affiliations:** 1 Department of Mechanical Engineering, College of Field Engineering, Army Engineering University, No. 1 Haifu Street, Nanjing 210007, Jiangsu, China; yangtiaotiaovv@163.com (X.Y.); baipanfeng1990@foxmail.com (P.B.); zxn8206@163.com (X.Z.); lizz0607@163.com (Z.L.); chenbb0708@163.com (L.C.); dafengyinqin@126.com (Q.Y.); 2 State Key Laboratory of Ultra-precision Machining Technology, Department of Industrial and Systems Engineering, The Hong Kong Polytechnic University, Kowloon 999077, Hong Kong, China; 3 State Key Laboratory of Disaster Prevention & Mitigation of Explosion & Impact, College of Defense Engineering, Army Engineering University, No. 1 Haifu Street, Nanjing 210007, Jiangsu, China

**Keywords:** gradient compressed porous metal, sound absorption performance, acoustic absorber, structural parameters, theoretical model, morphology characterization

## Abstract

Increasing absorption efficiency and decreasing total thickness of the acoustic absorber is favorable to promote its practical application. Four compressed porous metals with compression ratios of 0%, 30%, 60%, and 90% were prepared to assemble the four-layer gradient compressed porous metals, which aimed to develop the acoustic absorber with high-efficiency and thin thickness. Through deriving structural parameters of thickness, porosity, and static flow resistivity for the compressed porous metals, theoretical models of sound absorption coefficients of the gradient compressed porous metals were constructed through transfer matrix method according to the Johnson–Champoux–Allard model. Sound absorption coefficients of four-layer gradient compressed porous metals with the different permutations were theoretically analyzed and experimentally measured, and the optimal average sound absorption coefficient of 60.33% in 100–6000 Hz was obtained with the total thickness of 11 mm. Sound absorption coefficients of the optimal gradient compressed porous metal were further compared with those of the simple superposed compressed porous metal, which proved that the former could obtain higher absorption efficiency with thinner thickness and fewer materials. These phenomena were explored by morphology characterizations. The developed high-efficiency and thin-thickness acoustic absorber of gradient compressed porous metal can be applied in acoustic environmental detection and industrial noise reduction.

## 1. Introduction

The increasing noise pollution not only makes it one of the four major pollution problems all over the world, but also creates huge demand of the practical and excellent acoustic absorber, which make the development of sound absorbing materials one of focuses of the research in the fields of acoustics and environmental science [1,2,3,4,5]. Gwon et al. [1] had developed the flexible polyurethane foams with distinct cellular structures, and the high sound absorption ability was achieved by a high number of small cells. The lashing-structured nanofibrous aerogels were fabricated by Cao et al. [2], and average of the sound absorption coefficients at 250, 500, 1000, and 2000 Hz reached 0.41 when its density was 10.76 mg/cm^3^. Liu et al. [3] had proposed acoustical siphon effect for reducing thickness of the membrane-type metamaterials, and broadband absorption was obtained by a subwavelength six-unit sample in the low-frequency range of 400–650 Hz with the maximum absorption coefficient of almost 100% and the average absorption coefficient of about 80%. A sound absorbing metasurface with the coupled resonators was proposed by Li et al. [4], and over 99% energy absorption at a central frequency of 511 Hz with a 50% absorption bandwidth of 140 Hz was achieved experimentally. The microperforated dielectric elastomer actuator was proposed by Lu et al. [5], which obtained excellent sound absorption performance in the frequency range of 200–900 Hz. These novel acoustic absorbers can achieve the outstanding sound absorption performance, but the complicated absorbing structure, complex fabrication process, and finite absorption band limit their practical applications. Therefore, developing and preparing the high-efficiency and thin-thickness acoustic absorber from the mature sound absorbing materials remains an effective method at present.

Relative to the microperforated panel, the mature sound absorbing material of porous metal has the advantages of high strength, excellent fire resistance, outstanding machinability, and low fabrication cost, and many acoustic absorbers made of porous metals have been developed for noise reduction [6,7,8,9,10]. Porous metals with bottleneck type structures were proposed and optimized by Otaru et al. [6], and the optimal sound absorption performance in frequency range of 100–6000 Hz was obtained when its porosity was 0.68. The highly porous titanium foams had been investigated by Liu et al. [7], and its sound absorption coefficient could be more than 0.6 in the frequency range of 3150–6300 Hz and even exceed 0.9 at the resonance frequency. Open-cell IN625 foams with variable porosity and pore size were produced by Zhai et al. [8], which exhibited exceptional sound absorption properties of up to 0.95 at the resonance frequency and an overall sound absorption coefficient higher than 0.9 in frequency range of 1000–6000 Hz when its thickness was 50 mm. Jin et al. [9] had improved sound absorption performance of the open-celled aluminum foams by plasma electrolytic oxidation, and the experimental results indicated that the sound absorption increased gradually with the decrease of pore size or the rise of porosity. Review of sound absorption performance of the porous materials was summarized by Cao et al. [10], which included the organic foams, inorganic foams, and hybrid foams, and it could be concluded that there remained a huge challenge to fabricate porous materials with the high sound absorption coefficient in whole frequency range and the minimum thickness.

The gradient-structural porous metal and the multilayer porous metal have been developed to further improve absorption efficiency and reduce total thickness [11,12,13,14,15]. Zhu et al. [11] had prepared metal fiber porous material with gradient pore structures, and average sound absorption coefficient in the 50–6400 Hz for the optimal sample with thin-thickness of 3.0 mm reached 35%. Optimization of the gradient sintered metal fiber felts was presented by Meng et al. [12], and an outstanding sound absorption performance in the 500–1000 Hz range was achieved by the optimal acoustic absorber with the thickness of 10 mm. Chen et al. [13] had fabricated the multilayer porous fibrous metal for the sound absorption, and the average sound absorption coefficient in the 500–5000 Hz range reached 93.4% for the optimal four-layer porous fibrous metal with thickness of 50 mm. Cheng et al. [14] had developed the various nickel foam-based multilayer sound absorbing structure, and the optimal sound absorption coefficient could reach 0.4 in 1000–1600 Hz range for the composite structure of five-layer foam with backed 5-mm-thick cavity. The porous metal fiber materials with gradient structure was fabricated by Wang et al. [15], and its average absorption coefficient reached 37.52% in the 50–6400 Hz range when thickness of the three-layer gradient structure was 6 mm. It can be judged from this research that gradient and superposed porous metals will potentially realize high efficiency and thin thickness simultaneously.

It had been proved by Bai et al. [16] that sound absorption efficiency of the porous metal could be improved through compression, and the average sound absorption coefficient in the 1000–6000 Hz range reached 88.97% when the compression ratio was 70% and the thickness was 20 mm. It was also proved by Kádár et al. [17,18] that mechanical properties of the porous metal could be improved in the compression process through acoustic emission method. In order to further improve absorption efficiency and reduce total thickness, gradient compressed porous metal was prepared and studied in this research. Firstly, through deriving the structural parameters of thickness, porosity, and static flow resistivity of the porous metals after compression, the theoretical model of sound absorption coefficients of the gradient compressed porous metal was constructed by transfer matrix method [19,20,21] according to the modified Johnson–Champoux–Allard model [22,23,24]. Secondly, the original porous metal was compressed with the equidifferent compression ratios of 0%, 30%, 60%, and 90%, respectively, and the samples with different thickness, porosity, and static flow resistivity were obtained. Four-layer gradient compressed porous metals with different permutations were assembled, and their sound absorption coefficients in the 100–6000 Hz range were measured based on standing wave method [20,21,24,25]. Thirdly, sound absorption coefficients of the gradient compressed porous metal were compared with those of the simple superposed compressed porous metal with similar total thickness, which aimed to validate the sound absorption efficiency under the similar conditions. Finally, morphology characteristics of the single compressed porous metal sample with the different compression ratios were conducted by the scanning electron microscope, which aimed to provide the intuitive interpretations for its sound absorption performance.

## 2. Theoretical Modeling

In order to build a theoretical model of sound absorption coefficient of the gradient compressed porous metal, structural parameters of the single compressed porous material were derived firstly, which included the thickness d, porosity ϕ, and static flow resistivity σ, respectively.

(1) Thickness d

Compression ratio η was defined as the ratio of the reduced thickness by the compression to the original thickness. Therefore, thickness of the compressed porous metal d could be calculated by thickness of the original porous metal $d_{0}$ and compression ratio η, as shown in Equation (1).

(1) $$d=d_{0}\cdot \left(1-\eta \right)$$

(2) Porosity ϕ

For the original porous metal, supposing its porosity was $\phi_{0}$; its volume was $V_{0}$; volume of the metal frame structure in it was $V_{m0}$; volume of the pores that connected with the atmosphere in it was $V_{a0}$; its radius was $r_{0}$. Based on definition of the porosity [26], the relationships among these parameters are shown in the Equations (2), (3), and (4).

(2) $$\phi_{0}=\frac{V_{a0}}{V_{0}}$$

(3) $$V_{0}=V_{a0}+V_{m0}$$

(4) $$V_{0}=\pi r_{0}^{2}d_{0}$$

After the compression, it was considered that volume of the metal frame structure $V_{m0}$ and radius of the porous metal sample $r_{0}$ were not changed. For the compressed porous metal sample, supposing its volume was V; volume of the pores that connected with the atmosphere in it was $V_{a}$. The relationships among these parameters are shown in Equations (5), (6), and (7).

(5) $$\phi =\frac{V_{a}}{V}$$

(6) $$V=V_{a}+V_{m0}$$

(7) $$V=\pi r^{2}d$$

According to the relationships in Equations (1), (2), (3), (4), (5), (6), and (7), porosity of the compressed porous metal ϕ could be calculated, as shown in Equation (8). ϕ was decided by the original porosity $\phi_{0}$ and the compression ratio η together. Meanwhile, it could be found that $\phi \leq \phi_{0}$, which indicated that the porosity would be reduced by the compression.

(8) $$\phi =\frac{\phi_{0}-\eta }{1-\eta }$$

(3) Static flow resistivity σ

Calculation formula for static flow resistivity of the porous material is shown in Equation (9) [27]. Here, μ was viscosity of the air; $\sigma_{0}$ was static flow resistivity of original porous metal; $D_{0}$ was average aperture of the pores in the original porous metal.

(9) $$\sigma_{0}=\frac{32\mu }{\phi_{0}D_{0}^{2}}$$

After the compression, viscosity of the air μ was obviously not changed. The compression was along axis direction of the round porous metal sample, which was in accordance with incident direction of the plane wave in the measurement of sound absorption coefficient. Therefore, average aperture of the pores in the porous metal was in proportion to thickness of the sample, and it could be calculated by Equation (10) for the compressed porous metal.

(10) $$D=\frac{d}{d_{0}}D_{0}=\left(1-\eta \right)D_{0}$$

After the compression, the static flow resistivity σ could be calculated by Equation (11).

(11) $$\sigma =\frac{32\mu }{\phi D^{2}}$$

Based on the Equations (9), (10), and (11), the relationship between static flow resistivity σ of the compressed porous metal and the original static flow resistivity $\sigma_{0}$, original porosity $\phi_{0}$, and the compression ratio η could be obtained, as shown in Equation (12).

(12) $$\sigma =\frac{32\mu }{\phi D^{2}}=\frac{\phi_{0}\left(1-\eta \right)}{\phi_{0}-\eta }\sigma_{0}\cdot \frac{1}{{\left(1-\eta \right)}^{2}}=\frac{\phi_{0}}{\left(\phi_{0}-\eta \right)\left(1-\eta \right)}\sigma_{0}$$

According to the Equations (1), (8), and (12), structural parameters of the single compressed porous metal could be obtained. Based on the modified Johnson–Champoux–Allard model [20,21,22,23], transfer matrix P of the single compressed porous metal could be calculated by Equation (13). Here, k was wave number in it, which was obtained by Equation (14); Z was its characteristic impedance, which could be derived by Equation (15); j was symbol of imaginary number.

(13) $$P=\left[\begin{array}{cc} \cos\left(kd\right) & jZ\sin\left(kd\right)\\ jZ^{-1}\sin\left(kd\right) & \cos\left(kd\right) \end{array}\right]$$

(14) $$k=\omega \sqrt{\frac{\rho \left(\omega \right)}{K\left(\omega \right)}}$$

(15) $$Z=\sqrt{\rho \left(\omega \right)K\left(\omega \right)}$$

In Equations (14) and (15), ω was the sound angular frequency, which could be calculated by Equation (16); ρ(ω) was the complex effective density, which could be obtained by Equation (17); K(ω) was the complex effective bulk modulus, which could be achieved by Equation (18).

(16) ω=2πf

(17) $$\rho \left(\omega \right)=\rho \left[1+{\left(3^{2}+\frac{4\omega \rho }{\sigma \phi }\right)}^{-0.5}-j\frac{\sigma \phi }{\omega \rho }{\left(1+\frac{\omega \rho }{4\sigma \phi }\right)}^{0.5}\right]$$

(18) $$K\left(\omega \right)=\gamma P_{0}{\left[\gamma -\left(\gamma -1\right)\left(1-N_{u}{\left(j\frac{8\omega \rho P_{r}}{\sigma \phi }+N_{u}\right)}^{-1}\right)\right]}^{-1}$$

In Equation (16), f was the acoustic frequency. In Equation (17), ρ was density of the air under normal temperature, 1.21 Kg/m^3^. In Equation (18), *γ* was specific heat ratio of the air, 1.40; $P_{0}$ was standard static pressure of the air, 1.013 × 10^5^ Pa; $N_{u}$ was the Nusselt number, 4.36; Pr was the Prandtl number, 0.71 [16,21,28,29]. 

The original porous metals before compression were same in this study, which indicated that their original thickness, porosity, and static flow resistivity were same, and transfer matrix P of the single compressed porous metal was only determined by the compression ratio η. Supposing the corresponding transfer matrix was $P_{i}$ when compression ratio was $\eta_{i}$. According to transfer matrix method [19,20,21], Total transfer matrix T of the gradient compressed porous metal with n layers of single compressed porous metals was calculated by Equation (19), and the corresponding sound absorption coefficient α was obtained by Equation (20). Meanwhile, the corresponding total thickness could be calculated by Equation (21).

(19) $$T=\left[\begin{array}{cc} T_{11} & T_{12}\\ T_{21} & T_{22} \end{array}\right]=\prod_{i=1}^{n}P_{i}$$

(20) α=4Re(T11T21⋅1ρc)[1+Re(T11T21⋅1ρc)]2+[Im(T11T21⋅1ρc)]2

(21) $$TT=\sum_{i=1}^{n}d_{i}=\sum_{i=1}^{n}\left(1-\eta_{i}\right)d_{0}=\left(n-\sum_{i=1}^{n}\eta_{i}\right)d_{0}$$

**Figure 1:**
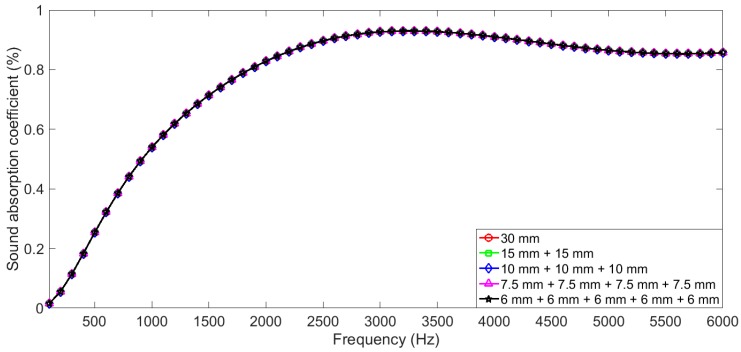
Comparisons of sound absorption coefficients of porous metal with different combinations.

In Equation (19), based on the transfer matrix method, each layer in the gradient compressed porous metal was treated as the individual, and the boundary conditions (from one layer to the next layer) was not taken into account, such as changes of the phase (the cosine terms) and the viscosity (the sine terms), reflection and transmission at the boundaries between the neighboring layers, and so on. Therefore, when structural parameters of thickness, porosity, and static flow resistivity of the porous metal were 30 mm, 0.95, and 10,200 Pa·s·m^−2^, respectively, influences of boundary conditions were validated through comparing theoretical sound absorption coefficients of the porous metals with the different combinations, and the selected boundary conditions were summarized in Table 1. Sound absorption coefficients of the porous metal in the 100–6000 Hz range were calculated according to Equation (19), and they are summarized in Figure 1. It could be observed that the obtained sound absorption coefficients were almost no different among the different combinations, which could further prove that transfer matrix method was effective in calculating sound absorption coefficients of the multilayer sound absorbers, and the boundary conditions had little effect.

If orders of single compressed samples in the gradient compressed porous metal were adjusted, the corresponding sound absorption coefficient would change accordingly. Therefore, influences of permutation of the single compressed porous metals with the different compression ratios to sound absorption performance of the four-layer gradient compressed porous metal were investigated in this study, which aimed to develop the novel acoustic absorber with high efficiency and thin thickness. Meanwhile, if each single sample in gradient compressed porous metal had the same compression ratio, it was named as the simple superposed compressed porous metal as the special case, and its sound absorption performance was also studied for contrast.

## 3. Materials and Measurements

**Figure 2:**
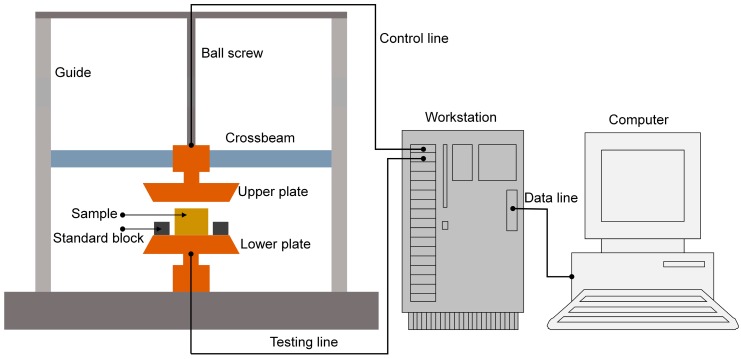
Schematic diagram of the CTM2050 universal testing machine for compression.

The original porous metal used in this study was porous copper (purchased from YiYang Foam metal New material Co., Ltd., Yiyang, Hunan, China). Its structural parameters of original thickness, porosity, and static flow resistivity were 5 mm, 0.95, and 10,200 Pa·s·m^−2^, respectively, which were provided by the supplier. The original porous metal sample was compressed by CTM2050 universal testing machine (Wuxi City Bleecker Trading Co., Ltd., Wuxi, Jiangsu, China) with the compression ratio from 10% to 90% at the interval of 10%, as shown in Figure 2. The original porous metal sample was placed on the lower plate. The standard block was used to ensure residual thickness of the compressed porous metal for certain compression ratio. The upper plate was fixed on the crossbeam, which could move on the ball screw along two guides on both sides. The mechanical system was connected to the workstation by control line and testing line. In order to avoid elastic deformation in the compression, compression force was set as 10 KN and compression process continued for 10 min.

**Figure 3:**
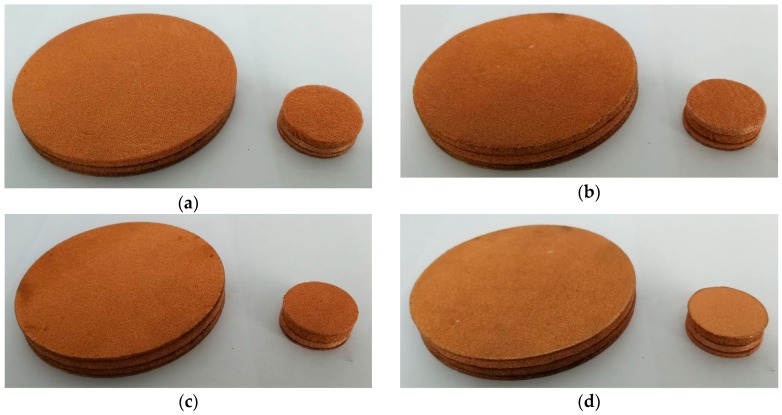
Representative assembled samples for the four-layer gradient compressed porous metals. (**a**) Sample serial of 001; (**b**) sample serial of 007; (**c**) sample serial of 013; (**d**) sample serial of 019.

**Figure 4:**
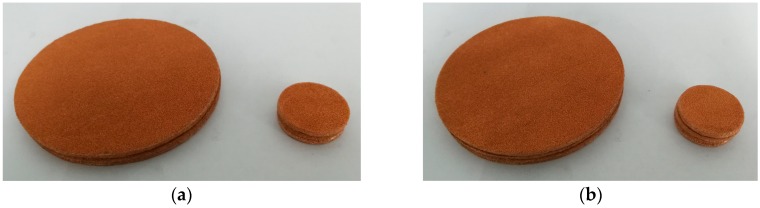
Representative prepared samples for the simple superposed compressed porous metals with the compression ratio of 40%. (**a**) With the thickness of 9 mm; (**b**) with the thickness of 12 mm.

After compression, four single samples with the equidifferent compression ratio of 0%, 30%, 60%, and 90% were assembled to prepare the four-layer gradient compressed porous metal with different permutations, as shown in Table 2. There were 24 permutations in total ($A_{4}^{4}=24$), which were numbered as 001 to 024 for sample serials, and some representative assembled samples were shown in Figure 3. Thickness of each four-layer gradient compressed porous metal was reduced from 20 mm to 11 mm, which could be obtained by Equation (21). According to the derived structural parameters of the single compressed porous metal in Equations (1), (8), and (12) and the constructed theoretical models in Equations (13), (14), (15), (16), (17), (18), (19), and (20), the theoretical average sound absorption coefficients in the 100–6000 Hz range were calculated, which were also summarized in Table 2. For each permutation of four-layer gradient compressed porous metal, its average sound absorption coefficient was obtained through averaging the corresponding sound absorption coefficient at each frequency point. For the 24 permutations, it could be found that maximum among the 24 average sound absorption coefficients was 67.47% when sample serial was 019 and minimum among the 24 average sound absorption coefficients was 40.75% when the sample serial was 001. Meanwhile, the simple superposed compressed porous metals with different compression ratios were also prepared when their total thicknesses were close to 11 mm, and the theoretical average sound absorption coefficients were calculated, which are summarized in Table 3. Meanwhile, some representative prepared samples for the simple superposed compressed porous metals were shown in Figure 4. Taking the simple superposed compressed porous metal with compression ratio of 40% in Figure 4 for example, the investigated thicknesses were 9 mm and 12 mm, which corresponded to original thicknesses of 15 mm and 20 mm before the compression, because thickness of the original single porous metal sample was 5 mm. It could be found that the optimal sound absorption performance was obtained for the simple superposed compressed porous metal with compression ratio of 70% when its total thickness was near 11 mm, which was consistent with the research results achieved by Bai et al. [16]. Although average sound absorption coefficient of the simple superposed compressed porous metal with the compression ratio of 70% reached 64.51% when its thickness was 10.5 mm, which was close to that 67.47% of the optimal four-layer gradient compressed porous metal with sample serial of 019, total thickness of the former before compression was 35 mm and that of the latter before compression was merely 20 mm. It could be concluded that the optimal four-layer gradient compressed porous metal could achieve higher sound absorption efficiency and utilize fewer materials relative to the simple superposed compressed porous metal when they had the similar total thicknesses. Meanwhile, through comparing the obtained theoretical average sound absorption coefficients in Table 2 and Table 3, it could be observed that not all sound absorption performances of the four-layer gradient compressed porous metals were better than those of the simple superposed compressed porous metals, which indicated that permutation of the four single compressed porous metals in four-layer gradient compressed porous metal was a critical factor.

**Figure 5:**
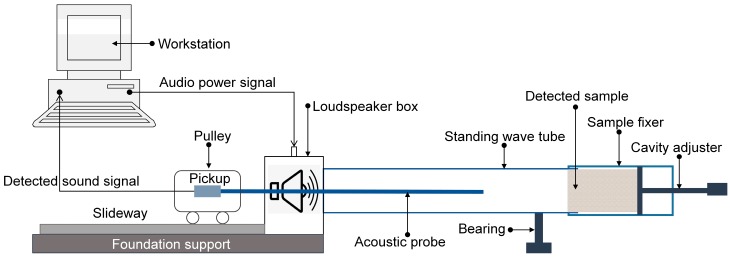
Schematic diagram of the AWA6128A detector to measure the sound absorption coefficient.

These prepared four-layer gradient compressed porous metals in Table 2 and simple superposed compressed porous metals in Table 3 were measured by the AWA6128A detector (Hangzhou Aihua instruments Co., Ltd., Hangzhou, Zhejiang, China), as shown in Figure 5, which could obtain sound absorption coefficient of the detected sample for certain sound frequency according to the standing wave method [29,30,31]. In order to facilitate the fair comparison, according to operating instruction of the AWA6128A detector, the measured frequency points in the low-frequency range were selected as 100 Hz, 200 Hz, 300 Hz, 400 Hz, 500 Hz, 600 Hz, 700 Hz, 800 Hz, 950 Hz, 1100 Hz, 1300 Hz, 1500 Hz, and 1800 Hz, which were obtained by measuring the samples with diameter of 96 mm; those in the high-frequency range were selected as 2000 Hz, 2300 Hz, 2600 Hz, 2900 Hz, 3200 Hz, 3500 Hz, 3800 Hz, 4100 Hz, 4400 Hz, 4700 Hz, 5000 Hz, 5300 Hz, 5600 Hz, and 6000 Hz, which were achieved by measuring the samples with diameter of 30 mm [16,20,24,29,31].

## 4. Results and Discussions

According to the Equations (1), (8), and (12), structural parameters of thickness, porosity, and static flow resistivity of the single compressed porous metal with different compression ratio could be calculated, as shown in the Table 4. It could be observed that the porosity was reduced, and the static flow resistivity was increased along with increase of the compression ratio. Although porosity and static flow resistivity could be adjusted by using different process parameters in the fabrication process, the adjustment was high-cost and time-consuming, especially in large-scale industrialized production, which indicated that compression could be considered as a simple and easy method to modify porosity and static flow resistivity of the already fabricated porous metal samples. In fact, the porous metal could not only be compressed, but also be stretched, which indicated that porosity and static flow resistivity of the porous metal could be easily adjusted by compression or stretching.

**Figure 6:**
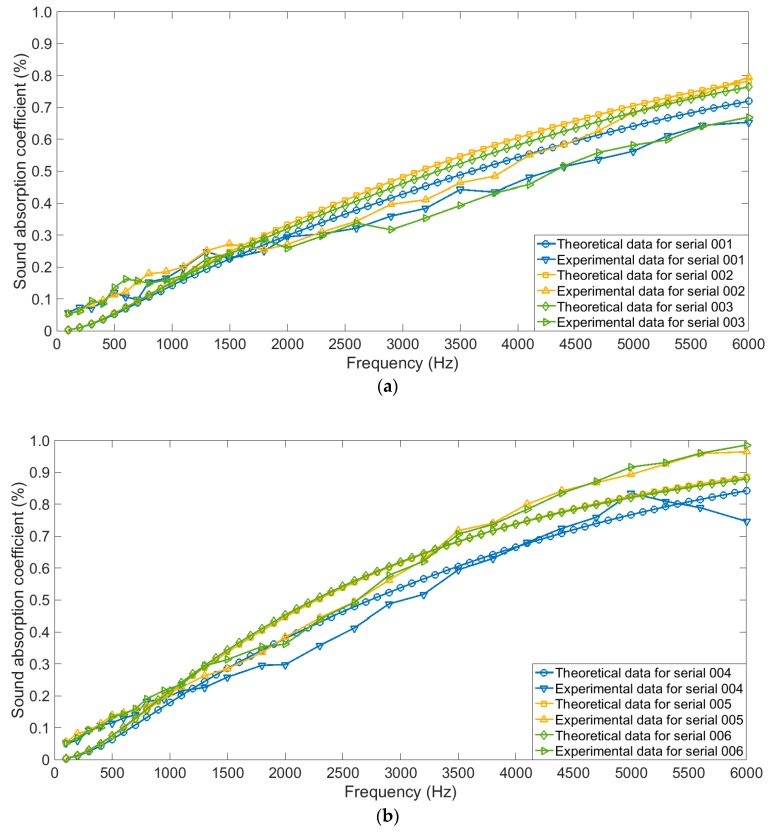

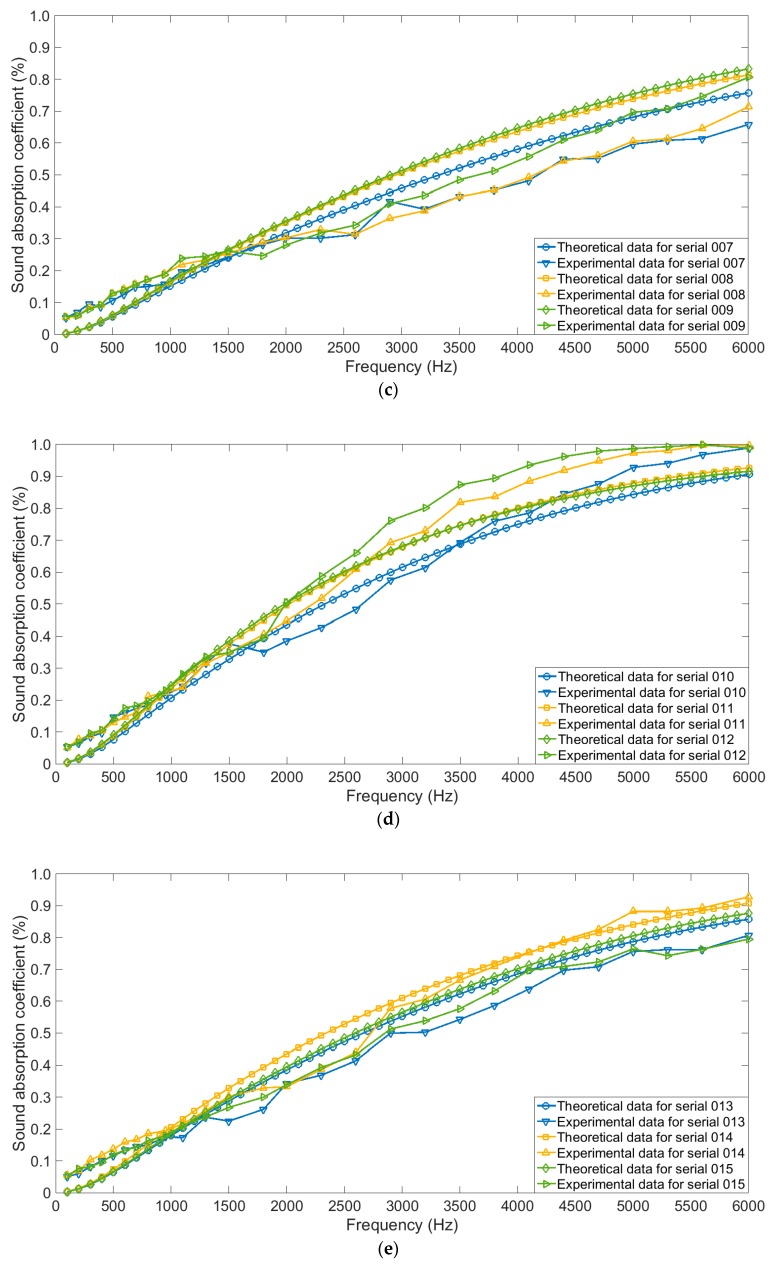

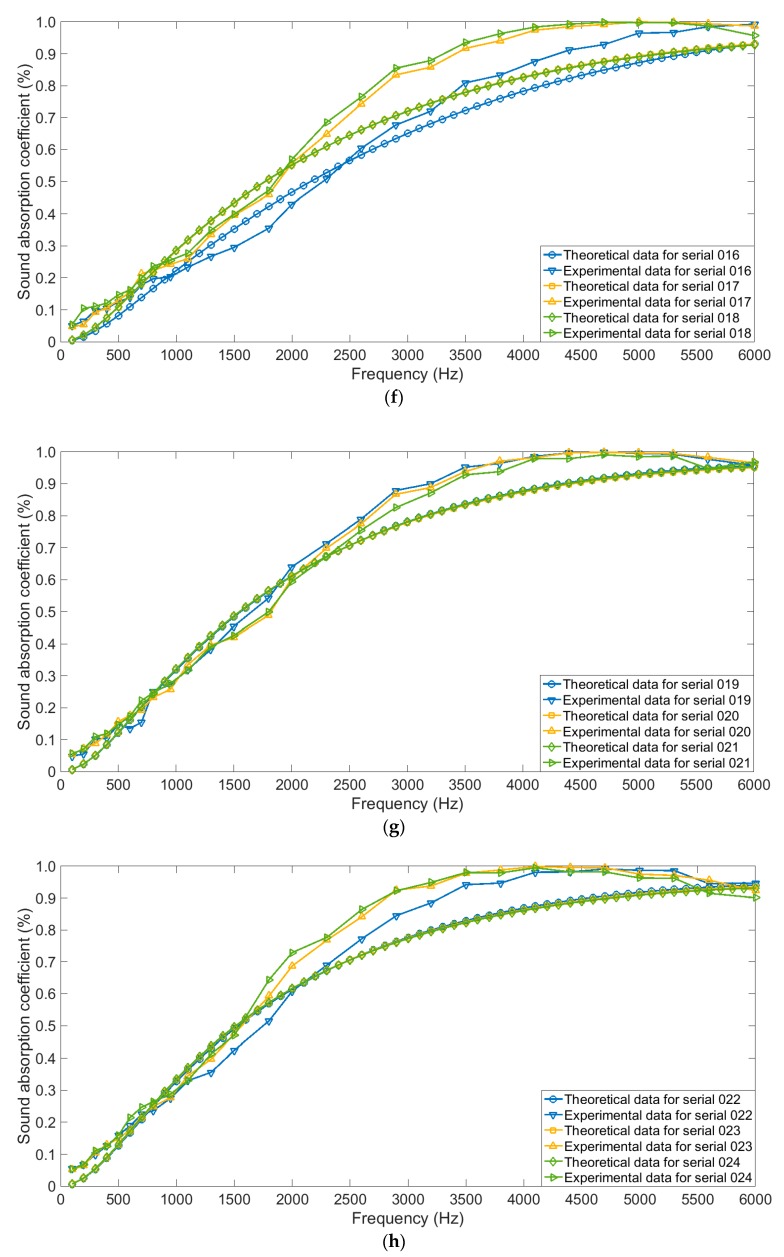
Comparisons of theoretical data and experiment data of sound absorption coefficients of the four-layer gradient compressed porous metals with different permutations. (**a**) Serials of 001–003; (**b**) serials of 004–006; (**c**) serials of 007–009; (**d**) serials of 010–012; (**e**) serials of 013–015; (**f**) serials of 016–018; (**g**) serials of 019–021; (**h**) serials of 022–024.

Comparisons of sound absorption coefficients of the four-layer gradient compressed porous metals with different permutations in theory and those in actual were shown in Figure 6. Every three groups of the results were put in one figure, which aimed to avoid the indistinguishability if too many results were placed. It could be judged from Figure 6 that variation trends of the sound absorption coefficients in actual were consistent with those in theory for each permutation of the four-layer gradient compressed porous metals. 

Meanwhile, comparisons of the calculated average sound absorption coefficients in actual and those in theory were shown in Table 5. It could be found that the optimal average sound absorption coefficient of 60.33% was obtained when sample serial was 019, and the corresponding compression ratios from first layer to fourth layer were 90%, 0%, 30%, and 60%, respectively. It was proved that this acoustic absorber could obtain high efficiency and thin thickness simultaneously.

**Figure 7:**
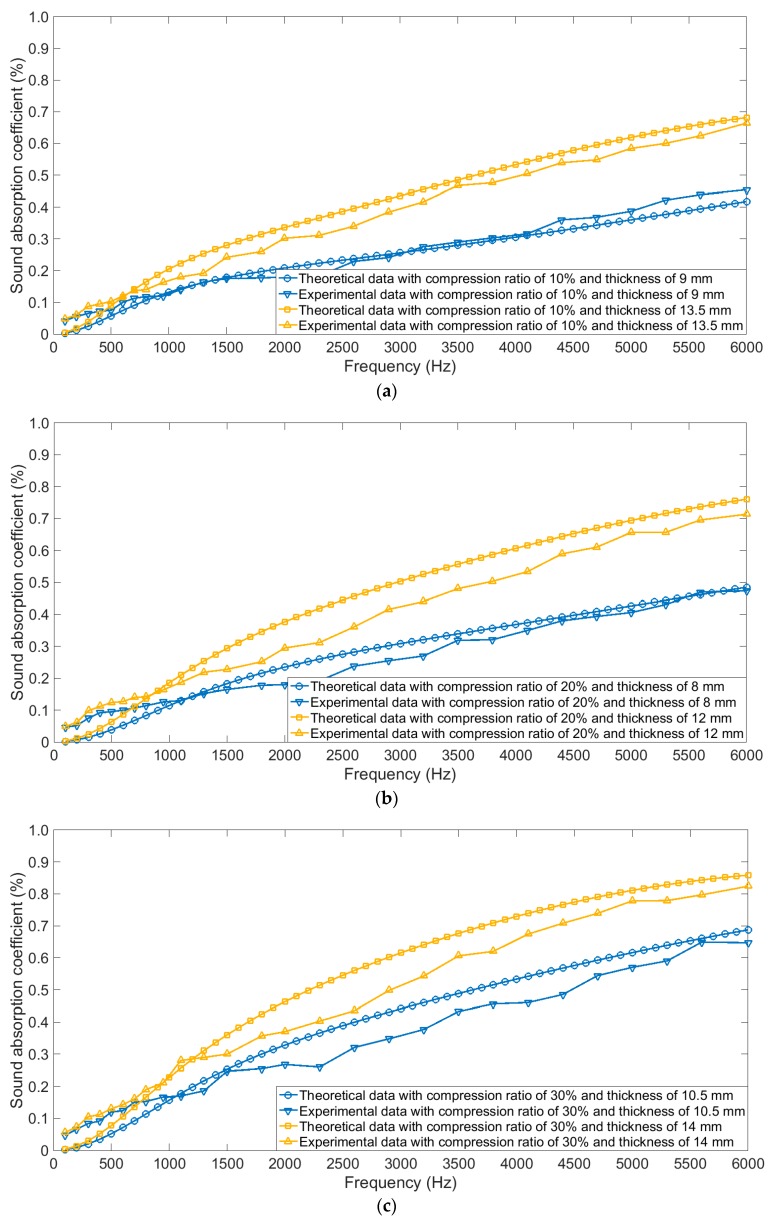

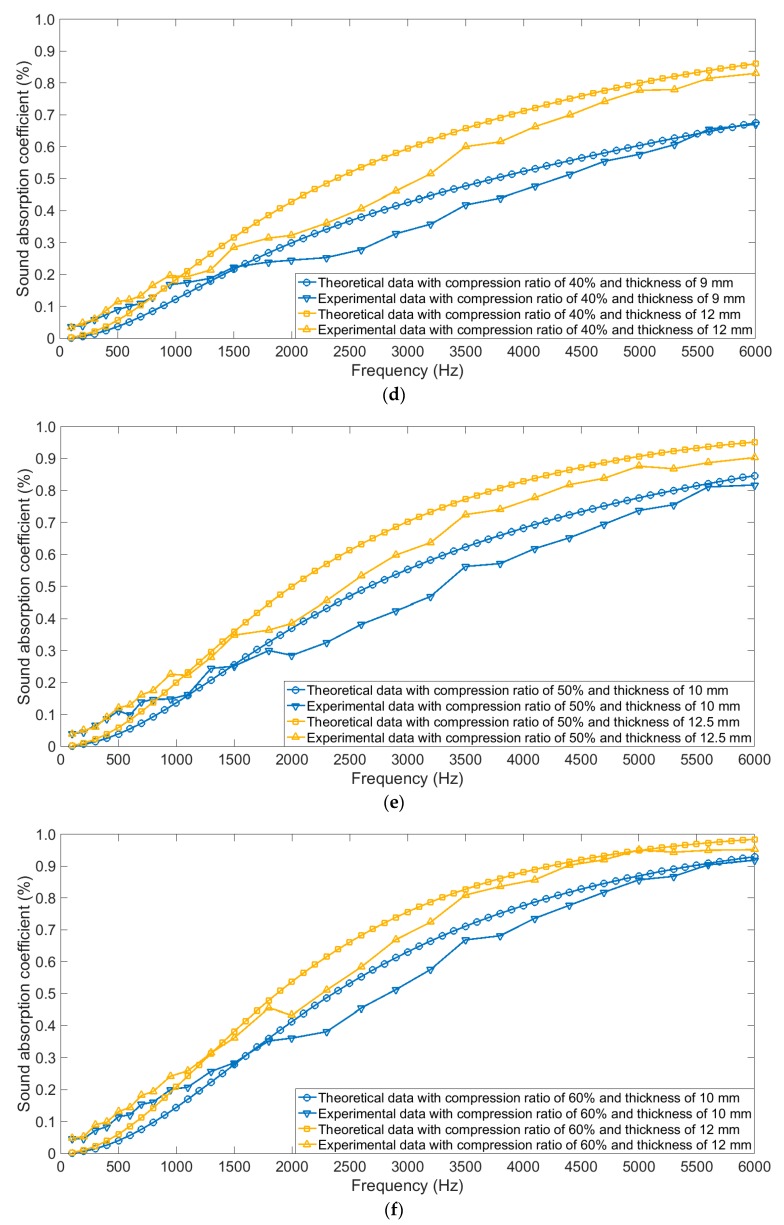

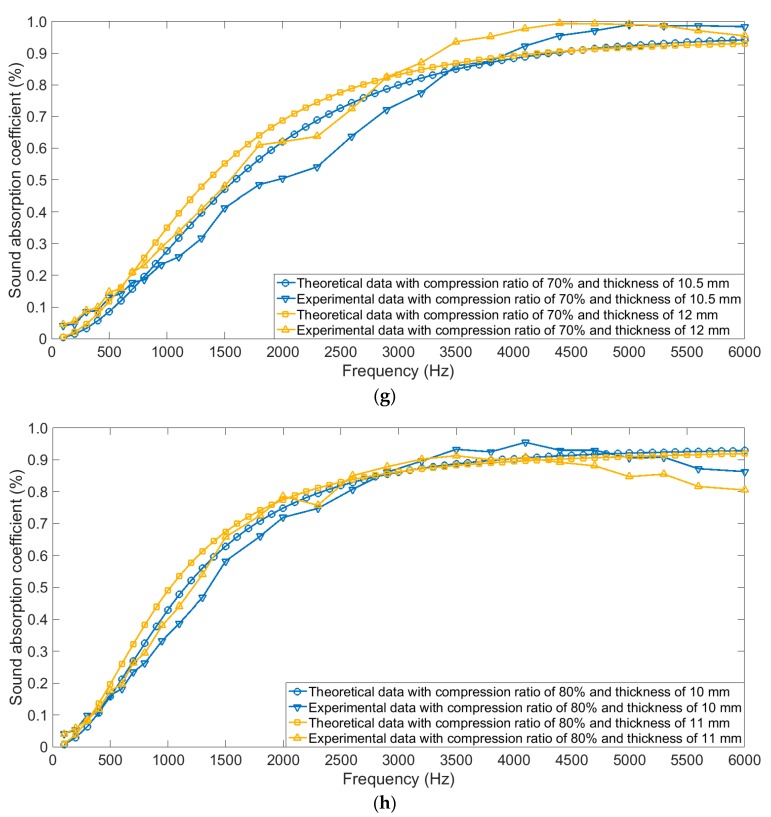
Comparisons of theoretical data and experiment data of sound absorption coefficients of the simple superposed compressed porous metals with different compression ratios. (**a**) 10%; (**b**) 20%; (**c**) 30%; (**d**) 40%; (**e**) 50%; (**f**) 60%; (**g**) 70%; (**h**) 80%.

When compression ratio ranged from 10% to 80%, comparative analyses of sound absorption coefficients of the simple superposed compressed porous metals in actual and those in theory were shown in Figure 7a–h, respectively. It could be observed that the theoretical data were consistent with the experimental data, which further proved effectiveness and accuracy of the derived structural parameters and constructed theoretical models. Meanwhile, it could be found that when the compression ratio was small, influence of thickness of the sample was notable, which could be judged from the Figure 7a–g. On the contrast, when the compression ratio reached 80% in Figure 7h, it could be found that distribution of sound absorption coefficient of the sample with the thickness of 10 mm was close to that of the sample with the thickness of 11 mm, no matter for the theoretical data or for the experimental data. The major reason for this phenomenon was that the sound absorption capability could easily reach its peak for the sample with thin thickness when the compression ratio was high enough.

Further quantitative comparisons of average sound absorption coefficients of the optimal four-layer gradient compressed porous metals and those of the simple superposed compressed porous metals with different compression ratios were summarized in Table 6. It could be observed that the optimal four-layer gradient compressed porous metal could obtain high average sound absorption coefficient of 60.33% with the thin thickness of 11 mm, and it used fewer original porous metals with thickness of 20 mm, which was obviously better than the simple superposed compressed porous metals with different compression ratios. Although the simple superposed compressed porous metal with the compression ratio of 70% achieved average sound absorption coefficient of 59.74% with thickness of 12 mm, its initial thickness before compression was 40 mm, which meant that it used more materials. Therefore, the optimal four-layer gradient compressed porous metal could be treated as a novel acoustic absorber.

## 5. Morphology Characterization

**Figure 8:**
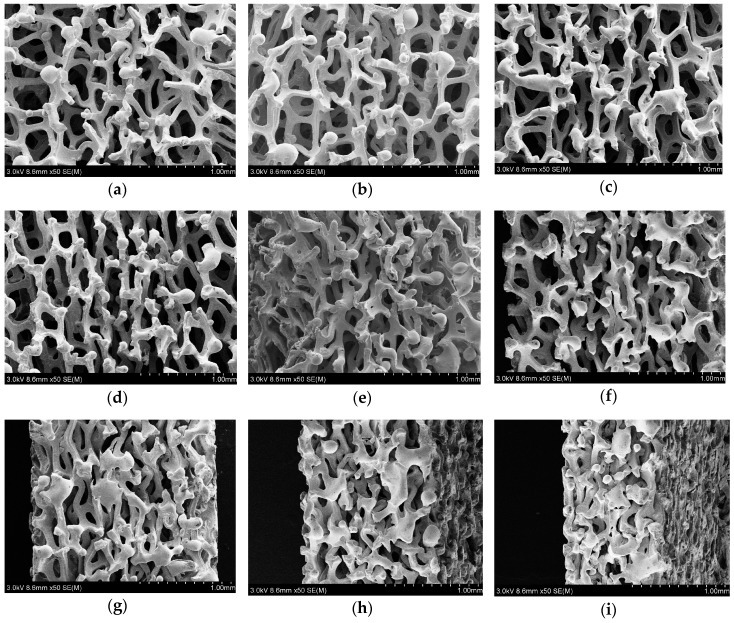
Section morphologies of the single compressed porous metals with different compression ratios. (**a**) 10%; (**b**) 20%; (**c**) 30%; (**d**) 40%; (**e**) 50%; (**f**) 60%; (**g**) 70%; (**h**) 80%; (**i**) 90%.

**Figure 9:**
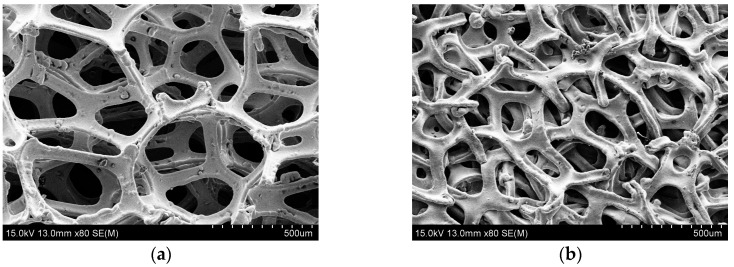
Comparisons of Surface morphologies of the porous metals. (**a**) Original sample before the compression; (**b**) compressed porous metal with compression ratio of 90%.

Section morphologies of the single compressed porous metal with the compression ratio ranged from 10% to 90% were detected by the scanning electron microscope (JSM-6360LV JEOL Ltd., Tokyo, Japan), and the obtained results were shown in Figure 8. The spherical solidified particles at the end of the exposed metal frame were not actual characteristics of the compressed porous metal, which were generated in melting the sample by the laser cutting [16]. It could be found that the pore structures in the porous metal were gradually flattened in the compression direction along with increase of the compression ratio, and appearance of the metal frame structure was almost not changed, which was consistent with the analysis process in deriving porosity and static flow resistivity of the compressed porous metal. The varied pore structures in the gradient compressed porous metal could be effective in absorbing the sound wave with different frequency range, which indicated that it was propitious to achieve better sound absorption performance comparing with the simple superposed compressed porous metal with the same or similar total thicknesses. Moreover, it could be judged from surface morphologies of the porous metals before and after compression in Figure 9 that average diameters of the pore structures in the original porous metal were 300–600 µm, and these standard polygonal pores were condensed to irregular micropores in the compressed porous metal. When compression ratio exceeded 90%, residual thickness of the compressed porous metal was less than 1 mm, which indicated that these micropores in the compressed porous metal could be partially considered as the microholes in the microperforated panel. That was also supposed as one reason for the achievement of optimal sound absorption performance for the four-layer gradient compressed porous metals with permutation of the compression ratio of 90% + 0% + 30% + 60%.

## 6. Conclusions

The sound absorption performances of four-layer gradient compressed porous metals and simple superposed compressed porous metals were studied in this research. Through theoretical modeling, experimental measurement, comparative analysis, and morphology characterization, the following conclusions were obtained.

(1) A high-efficiency and thin-thickness acoustic absorber was proposed through compression and assembly of the porous metal, and the prepared optimal four-layer gradient compressed porous metal could obtained the excellent average sound absorption coefficient of 60.33% in the 100–6000 Hz range with the thickness of 11 mm and permutation of the compression ratio of 90% + 0% + 30% + 60%.

(2) Sound absorption performance of the gradient compressed porous metal was remarkably influenced by permutation of the compression ratio. It could be observed that for 24 permutations of four-layer gradient compressed porous metals, the actual maximum and minimum among 24 average sound absorption coefficients obtained by the experimental measurements were 60.33% and 31.10%, respectively.

(3) Sound absorption performance of the optimal four-layer gradient compressed porous metal was better than that of the simple superposed compressed porous metal when total thickness of the acoustic absorber was same or similar, whether in theory or in actuality. Moreover, it could be judged from Table 6 that the optimal four-layer gradient compressed porous metal utilized fewer materials.

(4) For these investigated sound absorbing materials, consistence of the theoretical data and the experimental data validated effectiveness and accuracy of the derived structural parameters and the constructed theoretical models.

(5) Morphology characterization was conducted to explicate sound absorption performance of the prepared acoustic absorbers, which consisted of section morphology and surface morphology of the single compressed porous metal. The varied pore structures and the micropores in compressed porous metal with high compression ratio were considered as two major reasons for achievement of excellent sound absorption efficiency for the optimal four-layer gradient compressed porous metal. 

The prepared optimal four-layer gradient compressed porous metal could achieve higher sound absorption efficiency with thinner total thickness and fewer materials, which would promote the application of porous metal in acoustic environmental detection and industrial noise reduction.

## Figures and Tables

**Table 1:** Investigation on influences of the boundary conditions in calculating sound absorption coefficients of the porous metals with different combinations.

Number of Sample	Thickness of Each Layer (mm)					Number of Boundary
	1st Layer	2nd Layer	3rd Layer	4th Layer	5th Layer	
1	30	0	0	0	0	0
2	15	15	0	0	0	1
3	10	10	10	0	0	2
4	7.5	7.5	7.5	7.5	0	3
5	6	6	6	6	6	4

**Table 2:** The assembled four-layer gradient compressed porous metals with different permutations.

Sample Serials	Compression Ratio of the Single Sample				Theoretical Average Sound Absorption Coefficient (%)
	1st Layer	2nd Layer	3rd Layer	4th Layer	
001	0%	30%	60%	90%	40.75
002	0%	30%	90%	60%	45.04
003	0%	60%	30%	90%	43.54
004	0%	60%	90%	30%	49.81
005	0%	90%	30%	60%	55.32
006	0%	90%	60%	30%	55.42
007	30%	0%	60%	90%	43.33
008	30%	0%	90%	60%	47.26
009	30%	60%	0%	90%	48.12
010	30%	60%	90%	0%	55.76
011	30%	90%	0%	60%	59.96
012	30%	90%	60%	0%	59.99
013	60%	0%	30%	90%	50.95
014	60%	0%	90%	30%	55.48
015	60%	30%	0%	90%	52.17
016	60%	30%	90%	0%	58.43
017	60%	90%	0%	30%	63.15
018	60%	90%	30%	0%	63.10
019	90%	0%	30%	60%	67.47
020	90%	0%	60%	30%	67.27
021	90%	30%	0%	60%	67.43
022	90%	30%	60%	0%	67.08
023	90%	60%	0%	30%	66.96
024	90%	60%	30%	0%	66.84

**Table 3:** The simple superposed compressed porous metals with different compression ratios.

Compression Ratio	Thickness of Simple Superposed Sample		Theoretical Average Sound Absorption Coefficient (%)
	Before Compression (mm)	After Compression (mm)	
10%	10	9	29.15
	15	13.5	47.46
20%	10	8	28.58
	15	12	46.34
30%	15	10.5	44.68
	20	14	59.29
40%	15	9	42.23
	20	12	56.64
50%	20	10	52.65
	25	12.5	63.76
60%	25	10	58.19
	30	12	66.50
70%	35	10.5	64.51
	40	12	68.31
80%	50	10	59.21
	55	11	59.93

**Table 4:** Structural parameters of single compressed porous metal with different compression ratio.

Compression Ratio	Thickness (mm)	Porosity	Static Flow Resistivity (Pa·s·m^−2^)
0	5 ^1^	0.95 ^1^	10,200 ^1^
10%	4.5	0.9444	12,666.67
20%	4	0.9375	16,150
30%	3.5	0.9286	21,296.7
40%	3	0.9167	29,363.64
50%	2.5	0.9000	43,066.67
60%	2	0.8750	69,214.29
70%	1.5	0.8333	129,200
80%	1	0.7500	323,000
90%	0.5	0.5000	1,938,000

^1^ Provided by YiYang Foam metal New material Co., Ltd., Yiyang, Hunan, China.

**Table 5:** Comparisons of the calculated average sound absorption coefficients in actual and those in theory for the four-layer gradient compressed porous metals with different permutations.

Sample Serials	Compression Ratio of the Single Sample				Average Sound Absorption Coefficient (%)	
	1st Layer	2nd Layer	3rd Layer	4th Layer	In Actual	In Theory
001	0%	30%	60%	90%	31.11	40.75
002	0%	30%	90%	60%	34.83	45.04
003	0%	60%	30%	90%	31.10	43.54
004	0%	60%	90%	30%	39.66	49.81
005	0%	90%	30%	60%	46.23	55.32
006	0%	90%	60%	30%	46.57	55.42
007	30%	0%	60%	90%	31.84	43.33
008	30%	0%	90%	60%	32.69	47.26
009	30%	60%	0%	90%	35.58	48.12
010	30%	60%	90%	0%	47.19	55.76
011	30%	90%	0%	60%	51.28	59.96
012	30%	90%	60%	0%	53.82	59.99
013	60%	0%	30%	90%	38.13	50.95
014	60%	0%	90%	30%	44.31	55.48
015	60%	30%	0%	90%	39.56	52.17
016	60%	30%	90%	0%	50.06	58.43
017	60%	90%	0%	30%	56.07	63.15
018	60%	90%	30%	0%	57.23	63.10
019	90%	0%	30%	60%	60.33	67.47
020	90%	0%	60%	30%	58.18	67.27
021	90%	30%	0%	60%	59.89	67.43
022	90%	30%	60%	0%	57.92	67.08
023	90%	60%	0%	30%	57.65	66.96
024	90%	60%	30%	0%	57.29	66.84

**Table 6:** Comparisons of sound absorption efficiencies of the gradient compressed porous metals and those of the simple superposed compressed porous metals with the similar total thicknesses.

Sample Type	Compression Ratio	Thickness of Porous Metal Sample (mm)		Average Sound Absorption Coefficient (%)	
		Before Compression	After Compression	In Actual	In Theory
Optimal gradient compressed porous metal	90% + 0% + 30% + 60%	20	11	60.33	67.47
Simple superposed compressed porous metal	10%	10	9	21.72	29.15
	10%	15	13.5	31.80	47.46
	20%	10	8	22.63	28.58
	20%	15	12	33.94	46.34
	30%	15	10.5	30.63	44.68
	30%	20	14	41.41	59.29
	40%	15	9	29.63	42.23
	40%	20	12	39.07	56.64
	50%	20	10	36.80	52.65
	50%	25	12.5	45.56	63.76
	60%	25	10	43.00	58.19
	60%	30	12	50.41	66.50
	70%	35	10.5	59.03	64.51
	70%	40	12	59.74	68.31
	80%	50	10	57.63	59.21
	80%	55	11	58.12	59.93
